# Preparation of lactose-free pasteurized milk with a recombinant thermostable β-glucosidase from *Pyrococcus furiosus*

**DOI:** 10.1186/1472-6750-13-73

**Published:** 2013-09-21

**Authors:** Bin Li, Zemin Wang, Shiwu Li, William Donelan, Xingli Wang, Taixing Cui, Dongqi Tang

**Affiliations:** 1 Shandong University Qilu Hospital Research Center for Cell Therapy, Key Laboratory of Cardiovascular Remodeling and Function Research, Qilu Hospital of Shandong University, Jinan 250012, China; 2 Department of Pathology, Immunology, and Laboratory Medicine, University of Florida College of Medicine, Gainesville, FL 32610, USA; 3 Department of Cell Biology and Anatomy, University of South Carolina of Medicine, Columbia, SC 29209, USA

**Keywords:** Recombinant β-glucosidase, Thermostable enzyme, Pichia pastoris, Milk lactose hydrolysis, Pasteurization

## Abstract

**Background:**

Lactose intolerance is a common health concern causing gastrointestinal symptoms and avoidance of dairy products by afflicted individuals. Since milk is a primary source of calcium and vitamin D, lactose intolerant individuals often obtain insufficient amounts of these nutrients which may lead to adverse health outcomes. Production of lactose-free milk can provide a solution to this problem, although it requires use of lactase from microbial sources and increases potential for contamination. Use of thermostable lactase enzymes can overcome this issue by functioning under pasteurization conditions.

**Results:**

A thermostable β-glucosidase gene from *Pyrococcus furiosus* was cloned in frame with the *Saccharomyces cerecisiae* a-factor secretory signal and expressed in *Pichia pastoris* strain X-33. The recombinant enzyme was purified by a one-step method of weak anion exchange chromatography. The optimum temperature and pH for this β-glucosidase activity was 100°C and pH 6.0, respectively. The enzyme activity was not significantly inhibited by Ca^2+^. We tested the additive amount, hydrolysis time, and the influence of glucose on the enzyme during pasteurization and found that the enzyme possessed a high level of lactose hydrolysis in milk that was not obviously influenced by glucose.

**Conclusions:**

The thermostablity of this recombinant β-glucosidase, combined with its neutral pH activity and favorable temperature activity optima, suggest that this enzyme is an ideal candidate for the hydrolysis of lactose in milk, and it would be suitable for application in low-lactose milk production during pasteurization.

## Background

In vivo, lactase (β-D-galactosidase) is an enzyme secreted by intestinal villi that hydrolyses the disaccharide lactose into glucose and galactose [1,2] and is essential for the digestion of bovine milk [3] which contains an average of 4.8% lactose. Deficiency for this gene [3] leads to malabsorption of lactose and subsequent fermentation of lactose by the gut flora [4]. Lactose intolerance develops when afflicted individuals experience abdominal pain, diarrhea, bloating, flatulence, and other gastrointestinal symptoms following lactose consumption [5]. Avoidance of dairy products by lactose intolerant individuals often leads to insufficient calcium and vitamin D consumption and may cause adverse health outcomes, especially reduced bone mineral density and fractures [4,6].

Since lactose is a disaccharide composed of glucose and galactose, it can be hydrolyzed into these monosaccharides using either a β-glucosidase or β-galactosidase. Consumption of lactose-free milk, which can be produced by adding lactase enzyme (β-glucosidase or β-galactosidase) directly to milk, provides a means of maintaining good health while avoiding the symptoms of lactose intolerance. The lactase enzymes most commonly used in the industry are β-galactosidases, which are generally inhibited as glucose concentration increases. Lactase can be synthesized from many sources including animals, plants, bacteria, fungi, and yeast [7]. Although bacterial sources can efficiently produce lactase, they are not considered safe for use in food production due the risk of microbial contamination [8]. The lactase enzymes most widely used in industry are mesophilic enzymes from fungi (*Aspergillus spp.*) and yeast (*Kluyveromyces spp*.) [8]. Fungal sources, with acidic pH-optima, are effective for hydrolyzing lactose in whey, while yeast sources, with neutral pH-optima, are more effective for hydrolyzing lactose in milk [7]. Although pasteurization conditions denature many enzymes, several thermostable lactase enzymes have been identified from a variety of sources [9-12]. Hyperthermophilic archeaeon *Pyrococcus furiosus* harbors several hydrolytic enzyme activities; notably, it has a thermostable β-glucosidase (CelB) with a half-life of 85 h at 100°C. This enzyme can also function as a β-galactosidase, but the β-galactosidase activity is 34.3% of the β-glucosidase activity [10]. Although this enzyme has been cloned and functionally characterized in *Saccharomyces cerevisiae*, expression in batch culture produced low yields (~10 mg/L) which are not suitable for industrial applications [13]. Still, this β-glucosidase serves as a model system to use for lactose hydrolysis since it displays extreme stability and high catalytic activity in the presence of lactose.

In the present study, we cloned the *P. furiosus* β-glucosidase gene (CelB) into the pGAPZaA vector and electrotransformed it into the *Pichia pastoris* X-33 strain for large-scale production of heterologous proteins by high-density cell culture. The glyceraldehyde-3-phosphate dehydrogenase promoter (pGAP) has been used for large-scale constitutive expression of many heterologous proteins and eliminates problems associated with using the traditional AOX1 promoter such as the hazard and cost associated with the delivery and storage of large volumes of methanol [14-17]. We then analyzed the lactose hydrolytic action of this β-glucosidase under pasteurization conditions in order to establish a new method for the production of low lactose pasteurized milk. Our system provides a method for high level production of “food grade” thermostable lactase enzyme that is appropriate for use in the milk industry during the process of pasteurization, eliminating the need to add enzyme following pasteurization and eliminating the risk of microbial contamination.

## Results

### Fed-batch fermentation

**Figure 1:**
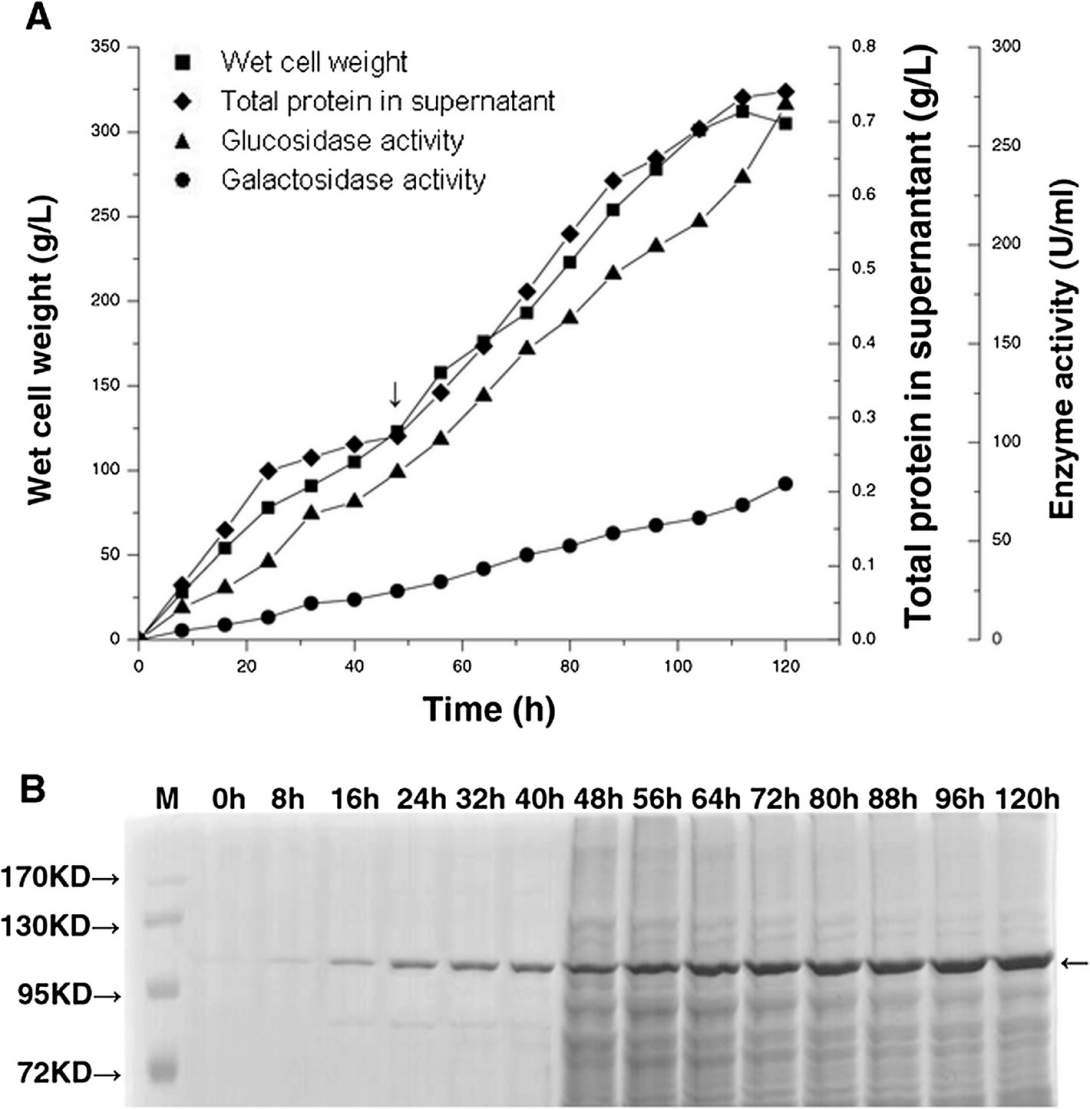
**Time course of recombinant enzyme properties and protein analysis during fermentation. A**. Wet cell weight (square), total protein in supernatant (diamond), β-glucosidase activity (triangle), and β-galactosidase activity (circle) were assessed as a function of time for 120 h fermentation in a 5 L fermenter. The arrow shows the time point for addition of glucose. **B**. Total protein in culture supernatant was assessed by Coomassie Brilliant Blue R-250 staining following SDS-PAGE (10%) during high cell density fermentation in a 5 L fermenter. Arrow at approximately 120 kDa shows the recombinant β-glucosidase. Each lane shows culture supernatant at indicated time and M = protein size marker.

Unlike the AOX1 promoter which requires methanol to initiate gene expression, the GAP promoter constitutively expresses genes in *P. pastoris* cells grown on many carbon sources [14-17]. Product formation appears to be growth associated in the GAP promoter constitutive expression system, therefore, a higher cell density would help to achieve higher product concentration. The fermentation was a fed-batch process carried out in 5 L fermenters. The X-33 cells were initially grown on basal salts medium and fed with 50% glucose after 48 h. Cell concentrations, total protein in supernatant, and enzyme activities during fermentation are shown in Figure 1A. The biomass accumulated at 120 h was 312 g/L, enzyme expression reached its peak with a yield of 740 mg/L, and the corresponding β-glucosidase activity was 27 1U/ml. We also tested the β-galactosidase activity of the enzyme and found it to be 94 U/ml, approximately 35% of the β-glucosidase activity. A protein corresponding to recombinant β-glucosidase, with a molecular mass of approximately 120 kDa, was detected upon performing SDS-PAGE analysis with Coomassie Brilliant Blue staining (Figure 1B). This band corresponds to the dimer unit of the enzyme. The native β-glucosidase is a tetramer, composed of four identical subunits, and each subunit has a molecular mass of 58 ± 2 kDa. The two monomer subunits dimerize by forming a disulfide bond and have a molecular mass of approximately 120 kDa. The enzyme dimers form a tetramer through non-covalent bonding, which is the functional native enzyme. It has been previously demonstrated that boiling in SDS is insufficient to denature the dimer which requires additional reagents, such as β-mercaptoethanol, to cleave the disulfide bonds.

### Purification of recombinant β-glucosidase

**Figure 2:**
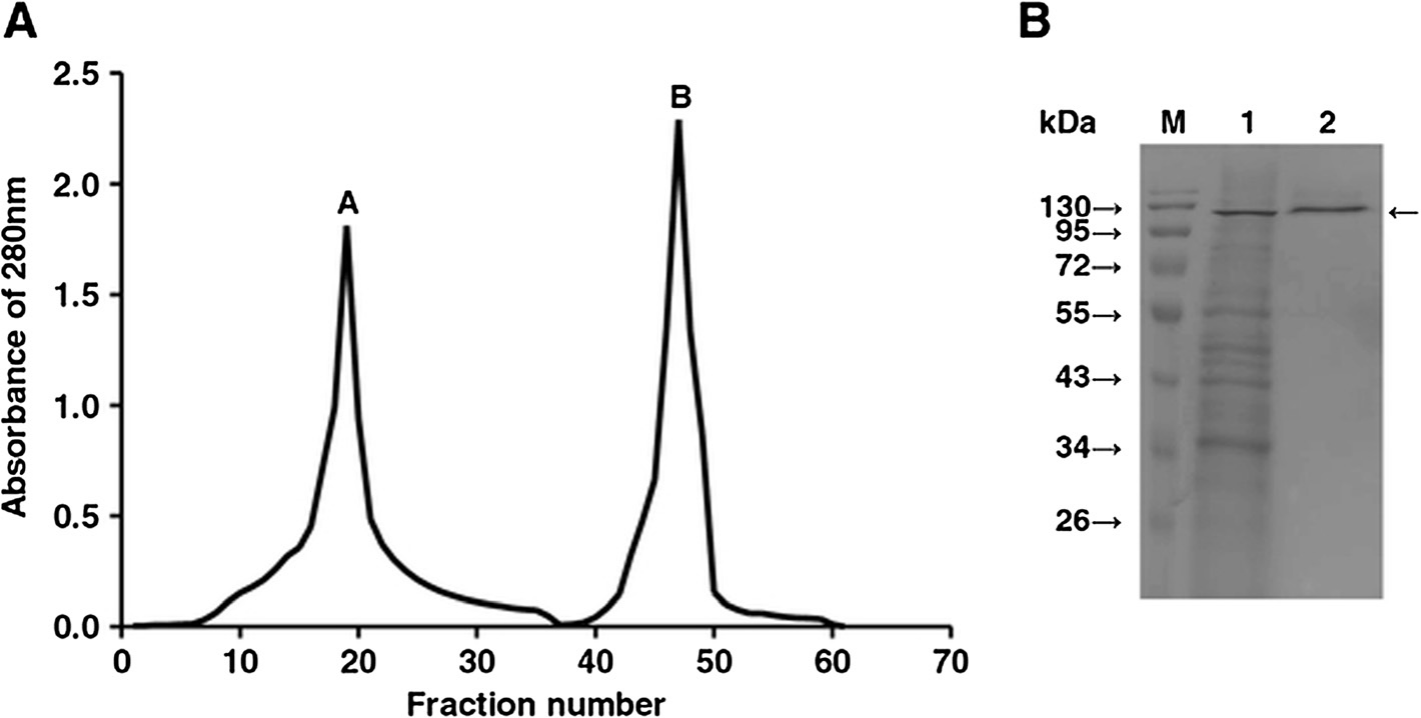
**DEAE anion exchange chromatography and SDS-PAGE analysis of the recombinant β-glucosidase. A**. Anion exchange chromatography was used to measure protein by its absorbance at 280 nm with increasing fractions as indicated on the x-axis. Two successive NaCl concentrations were used first to first elute non-specific protein and then to elute the recombinant β-glucosidase. Peak A demonstrates other impurity proteins eluted with 200 mM NaCl and peak B is recombinant β-glucosidase eluted with 400 mM NaCl. **B**. Following secretion, supernatant was collected and SDS-PAGE was performed before and after purification. Lane M shows a protein size marker, lane 1 shows total protein in culture supernatant before purification, and lane 2 shows recombinant β-glucosidase following purification. The arrow at approximately 120 kDa shows the β-glucosidase dimer.

Since the enzyme has a relatively low PI of 4.4 and few secreted proteins are present in the supernatant, we used one-step weak anion exchange chromatography to isolate the target protein, thus making the recombinant production of the enzyme more favorable. Samples of supernatant before and after purification were run side by side using SDS-PAGE (Figure 2B). Figure 2A shows the typical elution profile of chromatography. This procedure resulted in 1.9 fold purification and 80.8% recovery from the supernatant (Table 1).

**Table 1:** **Summary of the thermostable β-glucosidase purification from recombinant *Pichia pastoris***

**Step**	**Total protein (mg)**	**Total activity (U)**	**Specific activity (U/mg)**	**Yield (%)**	**Purification (fold)**
Cell-free extract	25.3	9362.3	366.1	100	1
DEAE anion exchange	10.9	7571.1	694.6	80.8	1.9

### Enzyme characterization

**Figure 3:**
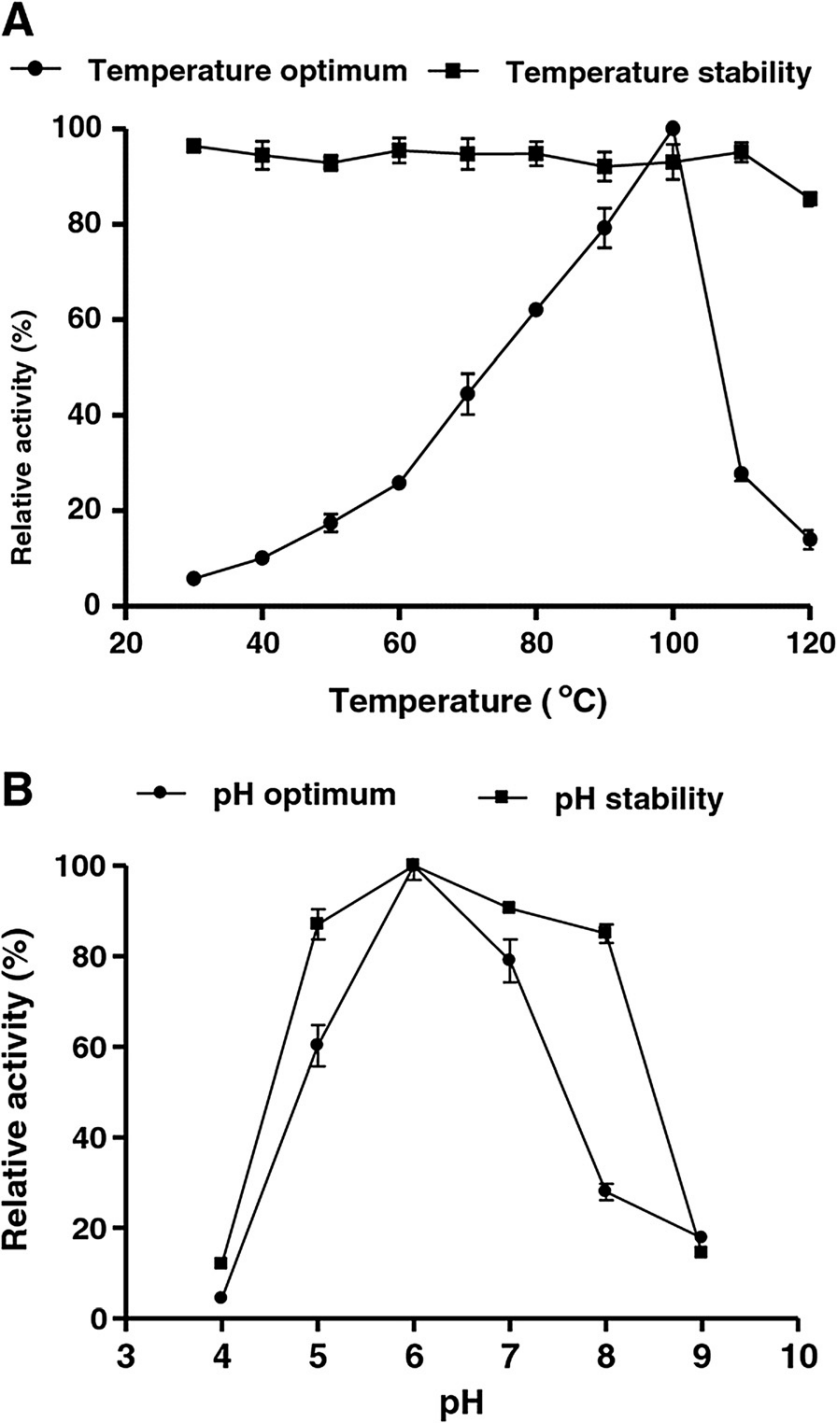
**Effects of temperature and pH on the recombinant β-glucosidase activity and stability. A**. β-glucosidase activities, as measured by temperature optimum (circle) or temperature stability (square) are shown as a function of temperature ranging from 30°C – 120°C. All data are presented as the mean ± SD of triplicate determinations. **B**. β-glucosidase activities, as measured by pH optimum (circle) or pH stability (square) are shown as a function of pH ranging from pH4 – pH9. All data are presented as the mean ± SD of triplicate determinations.

The effects of temperature on enzyme activity and stability are shown in Figure 3A. Optimum temperature was 100°C, and at 30°C the activity decreased to nearly 7% of the maximum activity. β-glucosidase was stable and retained more than 80% of its maximum activity from 30°C to 120°C. Regarding pH, β-glucosidase presented optimum activity at pH 6.0 (Figure 3B) and was nearly inactivated at extreme pH (pH 4.0 and 9.0). β-glucosidase retained more than 80% of its maximum activity from pH 5.0 to 8.0 when pre-incubated at 37°C for 1 hour. The effect of metal cations on the β-glucosidase activity is shown in Table 2, and although Cu^2+^ can reduce the relative activity to 78%, other metal cations have only negligible effects on the activity of the recombinant enzymes. Importantly, Ca^2+^ did not significantly inhibit the activity of the enzyme.

**Table 2:** Effects of metal ions on the activity of the recombinant β-glucosidase

**Metal ions**	**Relative activity ± SD (%)**
Control	100 ± 2.67
Mn^2+^	99.48 ± 3.54
Cu^2+^	77.67 ± 2.71
Na^+^	105.43 ± 3.89
Mg^2+^	102.26 ± 6.95
K^+^	100.95 ± 4.84
Ca^2+^	95 ± 4.76
Zn^2+^	98.67 ± 3.72

Effects of metal ions (1 mM) on the activity of the recombinant β-glucosidase are shown. Data are presented as the means ± SD of triplicate determinations.

### Hydrolysis of lactose in milk under pasteurization conditions

**Figure 4:**
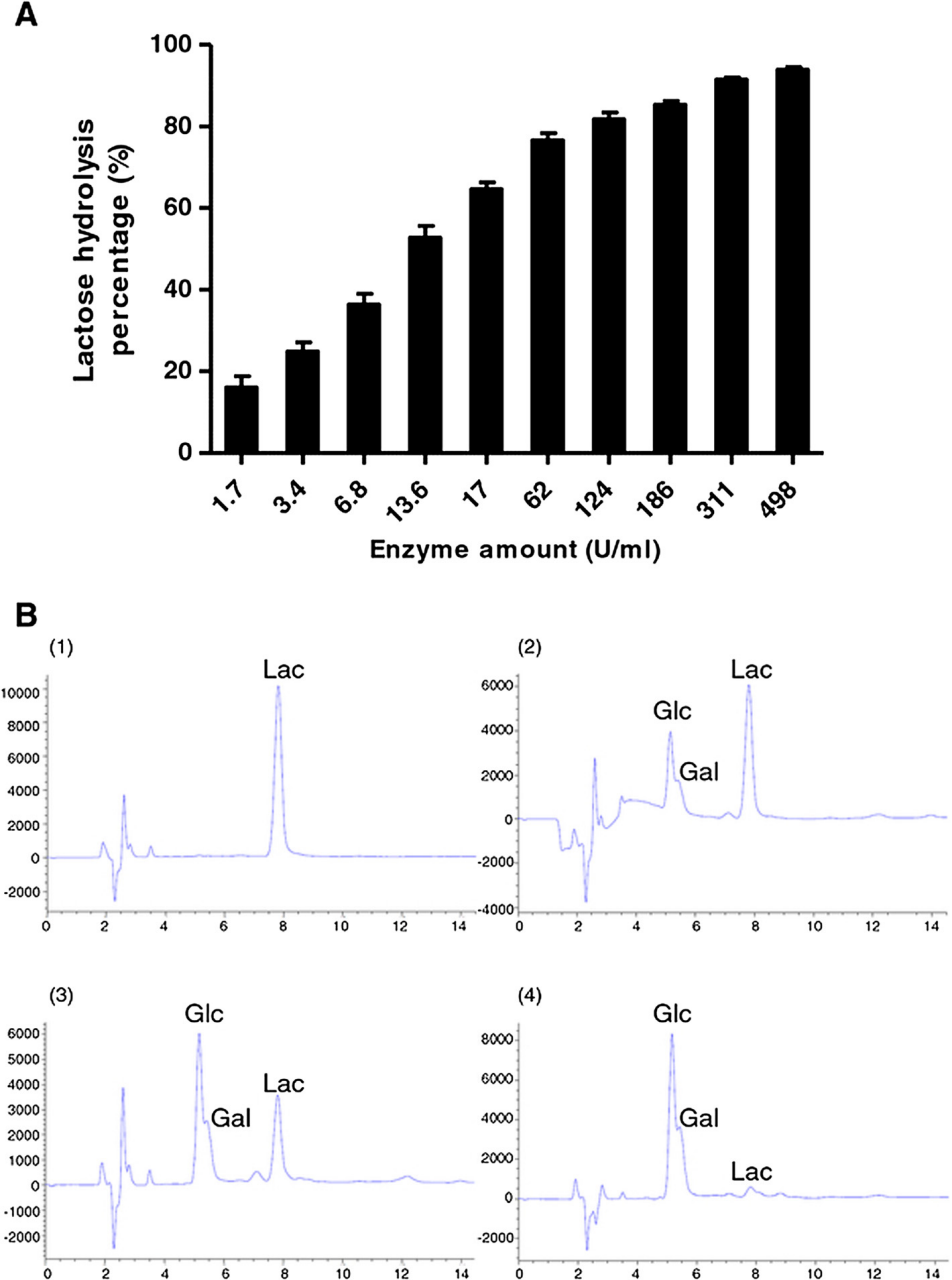
**Efficiency of β-glucosidase amount on lactose hydrolysis in milk and analyzed by HPLC. A**. Lactose hydrolysis was measured as a function of enzyme quantity (U/ml) at 65°C for 30 min using different concentrations of enzyme ranging from 1.7 U/ml to 498 U/ml as indicated. All data are presented as the mean ± SD of triplicate determinations. **B**. HPLC analysis of the reactions performed at 65°C for 30 min with 0 U/ml (Panel 1), 17 U/ml (Panel 2), 186 U/ml (Panel 3), or 498 U/ml (Panel 4) β-glucosidase enzyme. Lac = lactose, Glc = glucose, Gal = galactose.

**Figure 5:**
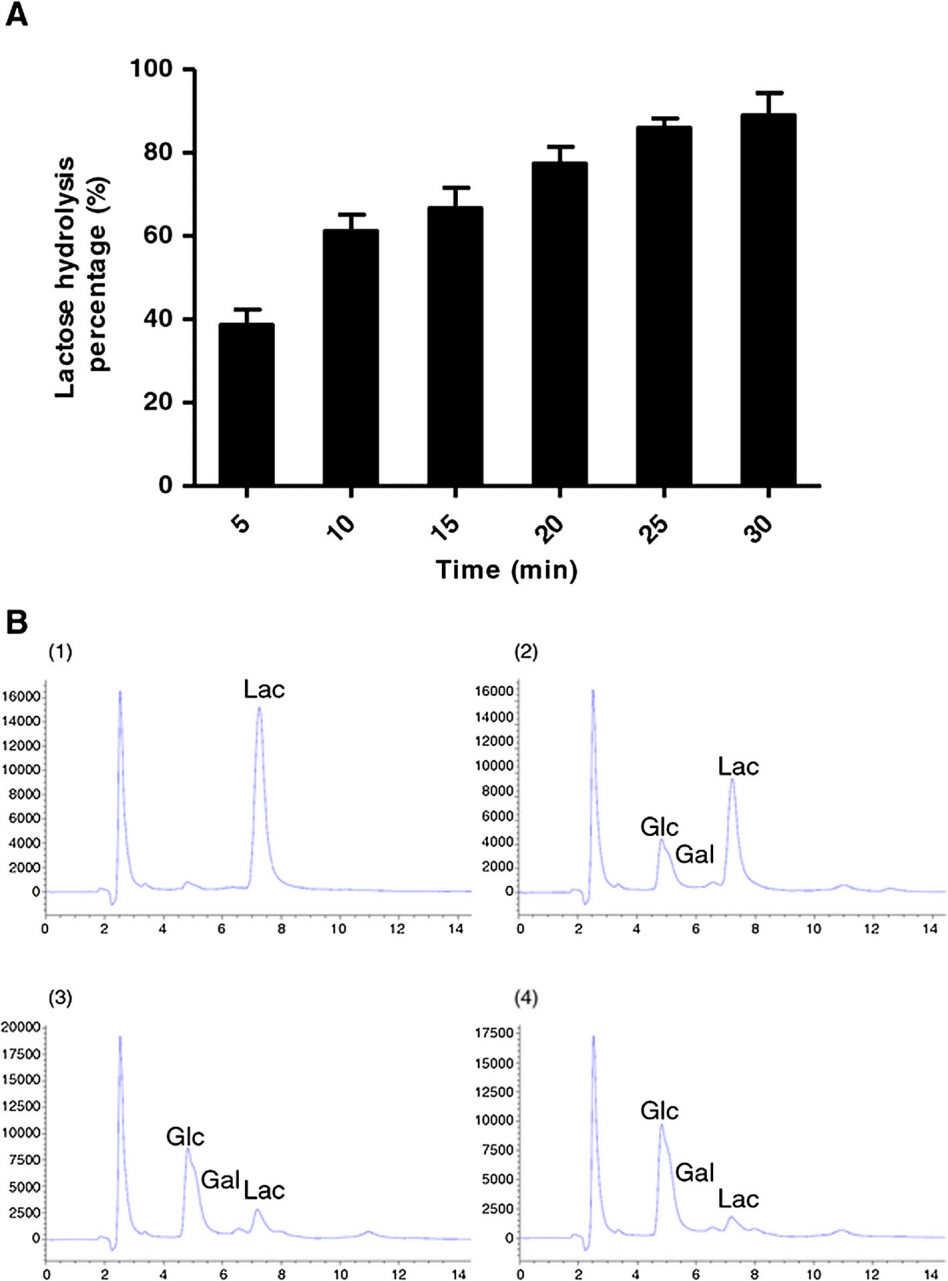
**Time course for efficiency of β-glucosidase on lactose in milk and analyzed by HPLC. A**. Lactose hydrolysis was measured as a function of time using 311 U/ml β-glucosidase enzyme at 65°C ranging from 5 min to 30 min. All data are presented as the mean ± SD of triplicate determinations. **B**. HPLC analysis of the reactions performed at 65°C using 311 U/ml β-glucosidase enzyme for 0 min (Panel 1), 10 min (Panel 2), 20 min (Panel 3), or 30 min (Panel 4). Lac = lactose, Glc = glucose, Gal = galactose.

**Figure 6:**
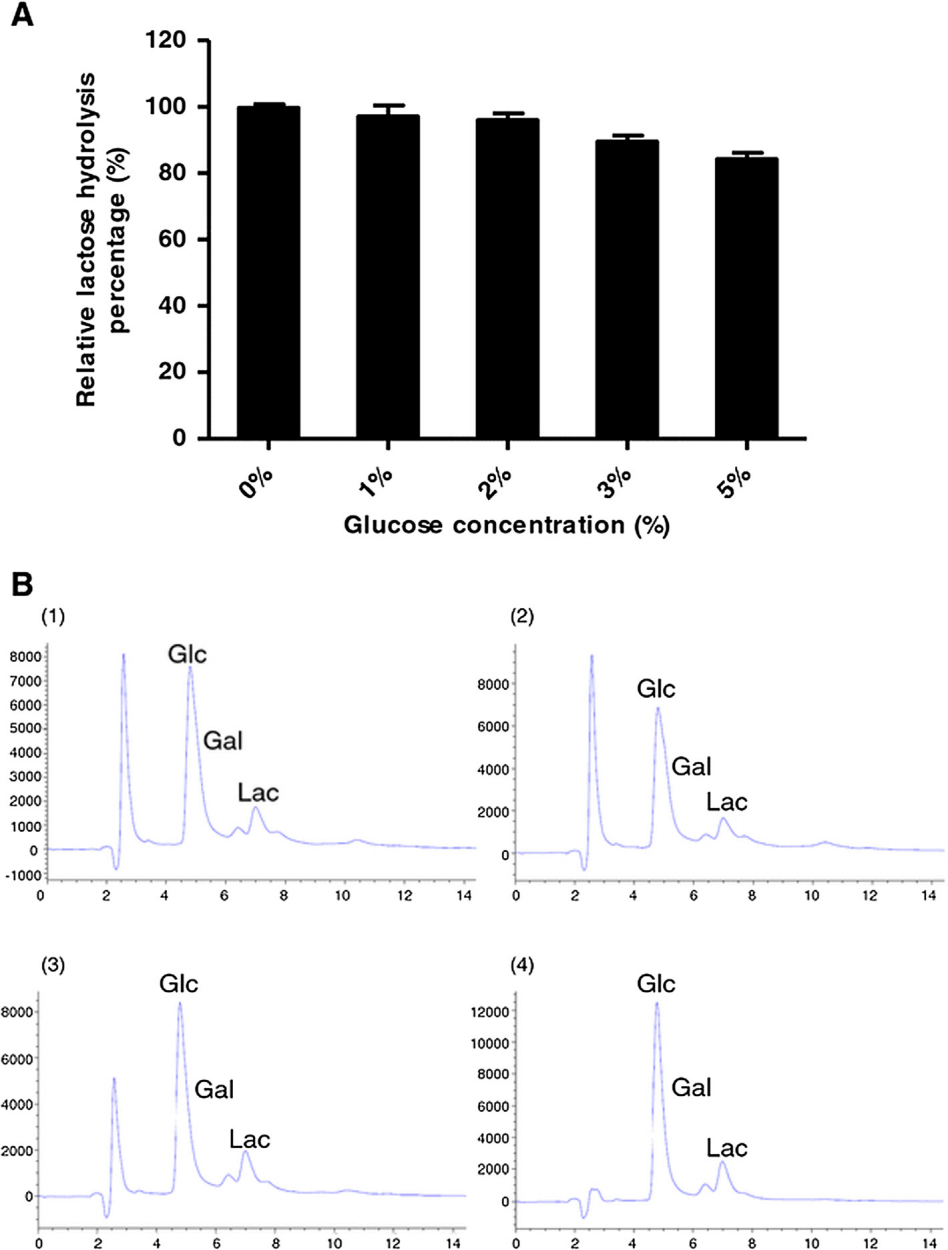
**Efficiency of β-glucosidase on lactose hydrolysis in milk with glucose. A**. Lactose hydrolysis was measured at 65°C for 30 min using 311 U/ml β-glucosidase enzyme with increasing concentrations of glucose ranging from 0-5% as indicated. All data are presented as the mean ± SD of triplicate determinations. **B**. HPLC analysis of the reactions performed at 65°C for 30 min using 311 U/ml β-glucosidase enzyme with the addition of 0% glucose (Panel 1), 1% glucose (Panel 2), 2% glucose (Panel 3), or 5% glucose (Panel 4). Lac = lactose, Glc = glucose, Gal = galactose.

The ability of β-glucosidase to hydrolyze the lactose in milk at 65°C for 30 min was tested using different amounts of the recombinant enzyme (Figure 4A) and then analyzed by HPLC (Figure 4B) which showed that the lactose in milk can be hydrolyzed into glucose and galactose. Using 17 U/ml of recombinant β-glucosidase achieved 50% hydrolysis and increasing to 498 U/ml of recombinant enzyme resulted in greater than 90% hydrolysis. We also tested the efficiency of hydrolysis of this enzyme (311 U/ml) from 5–30 minutes (Figure 5A-B) and found that after 20 minutes, the amount of hydrolysis was similar to 30 minutes. Therefore, we have demonstrated that the enzyme activity was more than sufficient to remove lactose in milk under pasteurization conditions. The effects of glucose concentration on hydrolysis was also tested, and we found that even high concentrations of glucose (5%) had minimal influence on the hydrolysis of lactose (Figure 6A-B), demonstrating that the enzyme had a relatively good tolerance to glucose. These results show that this recombinant thermostable β-glucosidase is highly-effective at hydrolyzing lactose in milk under pasteurization conditions.

## Discussion

Voorhorst [18] reported using *E. coli* to express the β-glucosidase gene. The expressed product remained within the host cell and necessitates complicated purification methodologies. Moreover, *E. coli* is not considered safe for food applications. Smith [13] reported expressing the β-glucosidase gene in *S. cerevisiae*, but the yield is very low at only 10 mg/L in batch culture. The development of the *S. cerevisiae* expression system was limited by the lack of a strong promoter and being incapable of high-density cell culture. Recently, *P. pastoris* has been widely used as an efficient expression system, secreting very low levels of its own proteins. The secreted heterologous protein is often virtually the only one protein in the culture medium. Thus, we cloned and expressed the β-glucosidase gene from *P. furiosus* into *P. pastoris*, and to our knowledge, this is the first use of *P. pastoris* to express this enzyme.

In the past, many researchers chose the methanol oxidase (AOX1) promoter to construct expression vectors for the production of a variety of recombinant proteins in *P. pastoris*[19-23]. It is especially effective for gene expression of products that are toxic to the host. However, methanol is a toxic substance, causes environment pollution, and is a fire hazard during production. Additionally, it is inconvenient to change from glucose or glycerol to methanol during fermentation. Therefore, we chose the constitutive GAP promoter for the production of this recombinant enzyme. This system is more desirable for large scale production because the hazard and cost associated with the storage and delivery of large volumes of methanol are eliminated.

The recombinant enzyme was cultivated in a 5 L fermentor, and after 120 h, we obtained 740 mg/L of β-glucosidase. Even when expression was not fully optimized, we achieved 700-fold higher levels than with *E.coli* expression and 70-fold greater than the *S. cerevisiae* system [13,18]. In another 300 L fermentor, we obtained 7 g/L of recombinant β-glucosidase after 160 h, which is quite convenient to use for industrial production.

The pH optimum of recombinant enzyme lies slightly above the β-glucosidase from *P. furiosus* (pH 5.0) and the temperature optimum (100°C) of the enzyme was similar with the β-glucosidase of *P. furiosus* (102-105°C). The purified enzymes showed a remarkable thermostability, and even incubation at 100°C for 1 hour didn’t change the activity significantly. The enzyme was not significantly stimulated or inhibited by divalent cations, as found for some glycosidases.

The ability of the recombinant β-glucosidase to hydrolyze lactose in milk under pasteurization conditions was tested and analyzed by HPLC. The lactose in the milk can be easily hydrolyzed into glucose and galactose. The maximum amount of hydrolysis was 91% when using 498 U/ml of the recombinant β-glucosidase at 65°C for 30 min. This is quite useful for industrial production of low lactose milk and milk powder under pasteurization conditions and the established industrial systems (which are much more efficient than those in our research laboratory) would likely require less enzyme to achieve similar results. In the past, the lactases most widely used in industry were obtained from *Aspergillus spp.* and *Kluyveromyces spp*, but they are all mesophilic enzymes, which cannot be used before pasteurization, and adding the enzymes after pasteurization leads to a high risk of contamination. Moreover, the hydrolysis time of these enzymes is generally more than 24 hours [24,25], which is too long for the production of pasteurized milk. However, these problems are all solved by using the recombinant thermotolerant β-glucosidase. Using this enzyme before pasteurization decreases the risk of bacterial contamination, and incubating at 65°C for 30 min can effectively hydrolyze more than 90% of milk lactose. In addition, we found the hydrolysis efficiency of the enzyme at 65°C for 20 minutes was similar to 65°C for 30 minutes. Different from many other glucosidases [26-30], glucose has relatively small influence on this enzyme. We added 5% glucose to the milk and found that the lactose hydrolysis efficiency only decreased slightly, highlighting another advantage of using this enzyme in low lactose milk production. In conclusion, the enhanced stability of this recombinant thermostable β-glucosidase, combined with its neutral pH activity and favorable temperature activity optima, suggest that this enzyme is an ideal candidate for the hydrolysis of lactose in milk and would be suitable for application in low-lactose milk production during milk pasteurization.

## Conclusions

This report is the first on the cloning, expression, purification, and characterization of the β-glucosidase from *P. furiosus* in *P. pastoris*. We achieved an enzyme yield of up to 740 mg/L using basal salts medium and purified the enzyme in one step anion exchange chromatography to near electrophoretic homogeneity. In this study, we established a new method to produce low lactose milk using this enzyme under pasteurization conditions. This method highlights the potential for low lactose milk production without additional steps required to add enzymes following pasteurization, and thus, decreases the risk of microbial contamination to a great extent.

## Methods

### Cloning and gene expression

The genomic DNA from *P. furiosus* DSMZ 3638 (ATCC), *P. pastoris* X-33, and pGAPZaA vector (Invitrogen) were used as the sources of the β-glucosidase gene (CelB), host cells, and expression vector, respectively. The gene encoding the β-glucosidase (CelB) was amplified by polymerase chain reaction (PCR) using the genomic DNA isolated from *P. furiosus* as a template. The sequence of the oligonucleotide primers used for gene cloning was based on the DNA sequence of *P. furiosus* β-glycosidase [Genbank accession number No. AF013169]. Forward primer [5’-GAATTCATGAAGTTCTCCAAAAAAC-3’] and reverse primers [5’-TCTAGACTTTCTTGTAACAAATT-3’] were designed to introduce the EcoRI and XbaI restriction sites (underlined), respectively, and were synthesized by Invitrogen. The amplified DNA fragment obtained by PCR was purified and inserted into the pGAPZaA vector digested with the same restriction enzymes. *Pichia pastoris* X-33 strain was transformed with the ligation mixture using an electroporator and plated on YPD agar containing 800 ug/ml of Zeocin (Invitrogen). A Zeocin-resistant colony was selected. Gene expression was evaluated by both sodium dodecyl sulfate polyacrylamide gel electrophoresis (SDS-PAGE) and enzyme activity.

### Fed-batch fermentation

Fermentation studies were conducted in 5 L fermenters (Baoxing Co, Shanghai, China). Inoculum was cultured in yeast extract peptone dextrose (YPD) medium. Cells were grown for 18 h at 30°C on a shaker at 220 rpm. Then, 10% (v/v) of the inoculum was inoculated into the 5 L fermenters containing 2 L basal salts medium. Basal salts medium used for fermenter batch cultivation contained (per liter deionized water): glycerol 40 g, H_3_PO_4_ (85%) 26.7 ml, K_2_SO_4_ 18.2 g, MgSO_4_.7H_2_O 14.9 g, KOH 4.13 g, CaSO_4_ 0.93 g, trace salts solution 4.35 ml (trace salts solution [per liter deionized water]: Fe_2_SO_4_.7H_2_O 65 g, ZnCl_2_ 20 g, CuSO_4_.5H_2_O 6 g, MnSO_4_.H_2_O 3 g, CoCl_2_ 0.5 g, Na_2_MoO_4_.2H_2_O 0.2 g, NaI 0.08 g, H_3_BO_3_ 0.02 g, Biotin 0.2 g, and H_2_SO_4_ 5.0 ml). The temperature was maintained at 30°C and the dissolved oxygen (DO) level was maintained between 25% and 35% by adjusting the agitation and aerating rate. The pH was kept at 6.0 by the addition of 28% (w/w) ammonium hydroxide. Foaming was controlled through the addition of antifoam. The glucose fed-batch feeding was started at the end of a batch phase triggered by the increase of DO above a set point of 50%. The composition of the fed-batch feeding medium was as follows (per liter deionized water): glucose 500 g/L and 1 ml PTM4 trace salts solution. Samples were taken at regular intervals and analyzed for biomass, total protein in medium, and enzyme activity.

### SDS-PAGE analysis

SDS-PAGE was performed using a 10% polyacrylamide gel on a vertical mini gel apparatus (Bio-Rad) at 120 V for 2 h. Protein molecular weight marker was purchased from Fermentas Life Science. Proteins were stained with Coomassie Brilliant Blue R-250.

### Purification of β-glucosidase

The yeast cells were removed by centrifugation at 8000 rpm for 10 min. The supernatant was filtered and loaded on diethylaminoethyl cellulose (DEAE)-sepharose weak anion exchange column (2.5 × 30 cm) equilibrated with 20 mM Tris–HCl buffer, pH 8.0. After washing with wash buffer (20 mM Tris–HCl, 200 mM NaCl), the β-glucosidase was eluted with 400 mM NaCl in the same buffer at a flow rate of 1 ml/min.

### Enzyme assay of the recombinant β-glucosidase

The colorimetric assay used to determine enzyme activity was modified from previously described protocols [25,28-30]. As a standard assay procedure, a standard curve was made using p-nitrophenol (pNP) in concentrations of 1 mM, and the absorbance was measured at 405 nm. Then, the β-glucosidase activity was measured at 85°C with 10 mM p-nitrophenol-β-D-glucopyranoside (pNPG, Sigma) as a substrate in 50 mM sodium citrate buffer (pH6.0). After 15 min of incubation, the reaction was stopped by adding 5% (w/v) Na_2_CO_3_ and the release of pNP was measured at 405 nm. The results were calculated by using the equation obtained in the standard curve. In all analyses, one unit (U) was defined as the amount of enzyme that releases 1 umol of pNP per minute. The β-galactosidase activity of the recombinant enzyme was measured at the same time using substrate ONP and ONPG with the same method.

### Effect of temperature on β-glucosidase activity and stability

In order to determine the optimum temperature of β-glucosidase, an appropriate concentration of the enzyme was incubated with 10 mM pNPG in 50 mM sodium citrate buffer (pH 6.0) for 15 min at different temperatures ranging from 30 to 120°C, and the activity was measured as the standard assay method. To determine the thermostability of β-glucosidase, the purified enzyme was pre-incubated at different temperatures ranging from 30°C to 120°C in 50 mM sodium citrate buffer (pH 6.0) without substrate. After incubating for 1 h, the residual β-glucosidase activity was measured according to the standard assay procedure using pNPG as the substrate and the maximum activity obtained was taken to be 100%. Data are presented as the means ± SD of triplicate determinations.

### Effect of pH on β-glucosidase activity and stability

The optimum pH value of β-glucosidase was determined by incubating the purified enzyme at 100°C for 15 min in different buffers in the range of 4.0–9.0. To determine the pH stability of β-glucosidase, the purified enzyme was incubated in different buffers in the range of 4.0–9.0 without substrate at 37°C for 1 h. The buffers used in this experiment were 50 mM sodium citrate, 50 mM sodium phosphate, and 50 mM glycine–NaOH. The remaining activities were measured according to the standard assay procedure using pNPG as the substrate and the maximum activity obtained was taken to be 100%. Data are presented as the means ± SD of triplicate determinations.

### Effect of metal ions on β-glucosidase activity

The effects of various metal ions (1 mM each of MnCl_2_, CuCl_2_, NaCl, MgCl_2_, KCl, CaCl_2_, and ZnCl_2_) on β-glucosidase activity were studied. The enzymes were individually pre-incubated with reagents at 37°C for 1 h. The remaining activities were measured as the standard assay procedures using pNPG as the substrate, and the control enzyme activity without cations was taken as 100%. Data are presented as the means ± SD of triplicate determinations.

### Hydrolysis of lactose in milk at the condition of pasteurization

The ability of β-glucosidase to catalyze lactose in milk at pasteurization condition was tested. 100 ml of raw milk was incubated with different amounts enzyme at 65°C for 30 min in a shaker at 120 rpm. Aliquots of 10 mL were removed at each time point (in time course study), transferred to 15-mL test tubes with screw-on caps, and immediately frozen at −80°C to stop the hydrolysis reaction. Lactose content was determined by HPLC. High-Performance Liquid Chromatography (HPLC) analysis of lactose hydrolysis was performed using an HPLC system with a quaternary pump, automatic injector, and differential refractive index detector (Agilent). The separation was carried out on a ZORBAX Eclipse XDB-NH_2_ column (5 um, 250 mm × 4.6 mm i.d.). The mobile phase was A (75% acetonitrile) and B (25% water). The flow rate was 1.4 ml/min, with an injection volume of 20 ul.

## Abbreviations

P. furiosus: Pyrococcus furiosus; E. coli: Escherichia coli; S. cerevisiae: Saccharomyces cerevisiae; PCR: Polymerase chain reaction; SDS-PAGE: Sodium dodecyl sulfate polyacrylamide gel electrophoresis; YPD: Yeast extract peptone dextrose; DO: Dissolved oxygen; DEAE: Diethylaminoethyl cellulose; pNP: p-nitrophenol; pNPG: p-nitrophenol-β-D-glucopyranoside; HPLC: High-Performance Liquid Chromatography.

## Competing interest

The authors declare that they have no competing interest.

## Authors’ contributions

BL carried out the fermentation, purification, enzyme characterization of the recombinant enzyme. ZMW participated in fermentation, purification and hydrolysis of lactose in milk. SWL participated in the cloning and expression of the β-glucosidase in *Pichia pastoris.* WD assisted with experimental analysis and manuscript editing. XLW and TXC contributed the study design and critical revision of the manuscript. DQT directed the cultivation of experiments and prepared the final manuscript. All authors read and approved the final manuscript.
